# The N-Terminal Domain and Glycosomal Localization of *Leishmania* Initial Acyltransferase *Lm*DAT Are Important for Lipophosphoglycan Synthesis

**DOI:** 10.1371/journal.pone.0027802

**Published:** 2011-11-17

**Authors:** Gada K. Al-Ani, Nipul Patel, Karim A. Pirani, Tongtong Zhu, Subbhalakshmi Dhalladoo, Rachel Zufferey

**Affiliations:** 1 Department of Biochemistry, Kansas State University, Manhattan, Kansas, United States of America; 2 Department of Biological Sciences, St. John's University, Jamaica, New York, United States of America; Federal University of São Paulo, Brazil

## Abstract

Ether glycerolipids of *Leishmania major* are important membrane components as well as building blocks of various virulence factors. In *L. major*, the first enzyme of the ether glycerolipid biosynthetic pathway, *Lm*DAT, is an unusual, glycosomal dihydroxyacetonephosphate acyltransferase important for parasite's growth and survival during the stationary phase, synthesis of ether lipids, and virulence. The present work extends our knowledge of this important biosynthetic enzyme in parasite biology. Site-directed mutagenesis of *LmDAT* demonstrated that an active enzyme was critical for normal growth and survival during the stationary phase. Deletion analyses showed that the large N-terminal extension of this initial acyltransferase may be important for its stability or activity. Further, abrogation of the C-terminal glycosomal targeting signal sequence of *Lm*DAT led to extraglycosomal localization, did not impair its enzymatic activity but affected synthesis of the ether glycerolipid-based virulence factor lipophosphoglycan. In addition, expression of this recombinant form of *Lm*DAT in a null mutant of *Lm*DAT did not restore normal growth and survival during the stationary phase. These results emphasize the importance of this enzyme's compartmentalization in the glycosome for the generation of lipophosphoglycan and parasite's biology.

## Introduction

Worldwide *Leishmania* parasites cause important human and animal diseases collectively called leishmaniasis. Disease transmission occurs upon biting by an infected female sand fly. The parasite develops extracellularly as flagellated promastigotes in the midgut of the insect vector, and intracellularly as non motile amastigotes within the phagolysosomal compartment of the vertebrate host's macrophages. *L. major* is responsible for the cutaneous form of leishmaniasis which manifests in a local self-healing skin lesion and affects approximately 1–1.5 million patients every year [1]. Ninety percent of cases of cutaneous leishmaniasis are found in Afghanistan, Pakistan, Syria, Saudi Arabia, Algeria, Iran, Brazil, and Peru [1].

Ether glycerolipids are major components of *Leishmania* membranes, representing approximately 20% of total cellular lipids [2], [3]. In *Leishmania major*, they are found primarily in the phosphatidylethanolamine and phosphatidylinositol glycerolipids [2], [3], [4], [5]. They are of particular importance for this parasite because ether glycerolipid based virulence factors such as lipophosphoglycan (LPG) and glycosylphosphatidylinositol-anchored proteins play critical roles throughout its life cycle (reviewed in [6], [7], [8], [9], [10]). Structurally, LPG is a complex glycolipid that is anchored to the plasma membrane *via* an ether lysophosphatidylinositol anchor [11] . The salient feature of LPG is the conserved domain consisting of the Galβ1,4Manα1-PO_4_ backbone of repeat units that in *L. major* are branched with galactose and arabinose residues [6], [9], [12], [13], [14].

**Figure 1:**
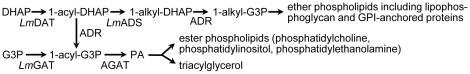
Glycerolipid biosynthetic pathways in *Leishmania*. AGAT, 1-acyl-glycerol-3-phosphate acyltransferase; ADR, alkyl/acyl-DHAP reductase; *Lm*ADS, alkyl-DHAP synthase; DHAP, dihydroxyacetonephosphate; *Lm*DAT, DHAP acyltransferase; *Lm*FAR, fatty acyl-CoA reductase; G3P, glycerol-3-phosphate; *Lm*GAT, G3P acyltransferase; PA, phosphatidic acid. Genes encoding ADR and AGAT in *Leishmania* are unknown.

In *Leishmania*, ether lipid biosynthesis initiates with the acylation of dihydroxyacetonephosphate (DHAP) by the DHAP acyltransferase (DHAPAT) *Lm*DAT, an obligatory step for the biosynthesis of ether lipids ([4], [15], [16]; Fig. 1). The product of this first acylation reaction, 1-acyl-DHAP, is then converted to 1-alkyl-DHAP by the alkyl DHAP synthase ADS1, which is further reduced to 1-alkyl-glycerol-3-phosphate (1-alkyl-G3P) by a NADPH-dependent alkyl/acyl-DHAP reductase [2], [17]. The intermediate 1-alkyl-G3P serves as the obligate precursor for all ether glycerolipids. Alternatively, in the absence of the G3P acyltransferase *Lm*GAT, 1-acyl-DHAP can be reduced to 1-acyl-G3P by a NADPH-dependent alkyl/acyl-DHAP reductase, which is subsequently used for the biosynthesis of ester glycerolipids [18]. The DHAPAT and alkyl-DHAP synthase are sequestered in the peroxisome-like organelle, called glycosome in *Leishmania* and related parasites [2], [16], [19], while the acyl/alkyl-DHAP reductase is associated with the glycosomes but its active site faces the cytoplasm [17].

*Lm*DAT is a unique DHAPAT that bears a very large N-terminal extension of approximately 650 amino acids that is absent in mammalian orthologs [16]. Our previous studies demonstrated that *Lm*DAT is important for growth, survival during stationary phase, the synthesis of ether lipids that includes the ether lipid based LPG, and for virulence, but is dispensable for raft formation [2], [4], [15]. All together, these data support the notion that *Lm*DAT may represent a potential target for anti-leishmanial chemotherapy. In the present work, a rational deletion approach was applied i) to address the role of the N-terminal extension of *Lm*DAT in enzyme stability and activity, and ii) to investigate the significance of *Lm*DAT glycosomal localization in the synthesis of the ether lipid based LPG. Last, point mutation analysis was carried out to assess whether a catalytically active *Lm*DAT enzyme is required to support normal growth and survival during the stationary phase of the parasite.

## Materials and Methods

### Strains and growth conditions

Promastigotes of *L. major* Friedlin V1 strain (MHOM/IL/80/Friedlin) were propagated in liquid and semi-solid M199-derived medium [2]. The null mutant *Δlmdat/Δlmdat* and complemented line *Δlmdat/Δlmdat [LmDAT NEO]* were described in [4]. Transfection was performed according to Ngo and colleagues [20] and selection was applied as appropriate in the presence of 20–40 µg/ml G418 or 25–50 µg/ml of hygromycin. To follow parasite proliferation, mid log phase parasites were diluted to 5×10^5^/ml and enumerated with a hemacytometer as a function of time.

### Plasmids

Deletion constructs of *LmDAT* were created by polymerase chain reaction (PCR) using pL-BSD.LmDAT [16] as a template, and the primer pairs O33 (‘5-CCGGGATCCCATATGAGCTTCCCACCACCTCGG-3’) and O116 (‘5- CGGGATCCTCACATCTTGGACAGAAGACGCTTTGCCCG-3’), O41 (‘5-CGGGATCCTCACATCTTGGATGGCTGTGTT-3’) and O111 (‘5-CGGGATCCATGCCCTATCACCAGTGTG-3’), and O41 and O136 (‘5-CGGGATCCATGACGGCGAACGGCTGGC-3’). The resulting amplified DNA fragments were digested with *Bam*HI, and ligated in sense orientation into the *Bam*HI sites of pXG.HV-LmDAT [16] to yield pXG.HV-LmDAT-ΔC_733_ (Ec395), pXG.HV-ΔN_546_-LmDAT (Ec569), and pXG.HV-ΔN_686_-LmDAT (Ec571), respectively.

The plasmid for the expression of a truncated form of *Lm*DAT, lacking the C-terminal glycosomal targeting tripeptide SKM (*Lm*DAT-ΔC_3_) was generated by PCR using the oligonucleotides O34 (‘5-CCGGGATCCCATATGAGCTTCCCACCACCTCGG-3’) and O112 (‘5-CGGGATCCTCATGGCTGTGTTAGCTCACGG-3’), and pL-BSD.LmDAT as a template. The obtained DNA fragment was subsequently digested with *Bam*HI and cloned as a 4.3 kb fragment in sense orientation into the respective sites of pXG.HV-LmDAT to give pXG.HV-LmDAT-ΔC_3_ (Ec440).

The K852L mutant form of *Lm*DAT was created by introducing the point mutation by PCR using oligonucleotides O133 (‘5-AGGAAAGCTTCATAAAGAGGGCGCCGCTG-3’) and O134 (‘5-TATGAAGCTTTCCTTCCTTCCGCGACGACCCG-3’), and pL-BSD.LmDAT as a template. In addition, a *Hin*dIII site was introduced to “tag” the mutation. The mutated fragment was swapped with the corresponding wild-type DNA using the *Sac*I and *Mlu*I sites of pBEVY-L.LmDAT described in [16] to give pBEVY-L.LmDAT^K852L^ (Ec276). This plasmid was subsequently digested with *Bam*HI and the excised 4.3 kb fragment was ligated in sense orientation into the respective sites of pXG.HV-LmDAT [16] to yield pXG.HV-LmDAT^K852L^ (Ec456). All amplified DNAs were verified by sequencing.

The plasmids pXG.HV-LmDAT [16], pXG.HV-LmDAT-ΔC_733_, pXG.HV-ΔN_546_-LmDAT, pXG.HV-ΔN_686_-LmDAT, pXG.HV-LmDAT^K852L^, and pXG.HV-LmDAT-ΔC_3_ were transformed into the null mutant *Δlmdat/Δlmdat* to give the strains *Δlmdat/Δlmdat [HV-LmDAT NEO]*, *Δlmdat/Δlmdat [HV*-*LmDAT-ΔC_733_ NEO]*, *Δlmdat/Δlmdat [HV-ΔN_546_-LmDAT NEO], Δlmdat/Δlmdat [HV-ΔN_686_-LmDAT NEO], Δlmdat/Δlmdat [HV-LmDAT^K852L^ NEO],* and *Δlmdat/lmdat [HV-LmDAT-ΔC_3_ NEO]*, respectively. In addition, pXG.HV-LmDAT and pXG.HV-LmDAT-ΔC_3_ were also transformed into the wild type to yield FV1 *[HV-LmDAT NEO]* and FV1 *[HV*-*LmDAT-ΔC_3_ NEO]*, respectively.

### Enzymatic assays

*Leishmania* protein extracts were prepared as described previously [16], [18]. Protein concentration was determined by the bicinchoninic acid assay using bovine serum albumin as a standard. DHAPAT activity was assessed by measuring the acylation rate of DHAP produced by catabolism of fructose-1,6-biphosphate by the action of an aldolase and a triose phosphate isomerase, based on a protocol established by Bates and Saggerson as described in [16]. The specificity of [U-^14^C]D-fructose-1,6-biphosphate (MP Biomedicals) was 295 mCi/mmol.

### Digitonin fractionation and electrophoresis

For digitonin treatment fresh end-log cells were harvested, washed once in phosphate buffered saline (PBS), and resuspended in 20 mM TrisHCl (pH 8.0), 1 mM EDTA, 1 mM DTT containing a protease inhibitor cocktail (Roche) at a cell density of 2×10^8^/ml. Aliquots of 100 µl were made and supplemented with increasing (0 to 0.6 mg/ml) concentrations of digitonin (stock solution of 15 mg/ml in PBS) and incubated at 26°C for 10 min. Cells were then centrifuged at 20,800 g for 2 min. Supernatants were immediately removed and resolved by sodium dodecyl sulfate polyacrylamide gel electrophoresis (SDS-PAGE).

Western blot analyses in the presence of monoclonal WIC79.3 (specific to β-galactose side-chains; generous gift from S. Turco; [21]) and V5 (Invitrogen) antibodies, and polyclonal immunoglobulins specific to hypoxanthine guanine phosphoribosyltransferase (generous gift from A. Jardim; [22]), phosphomannomutase (generous gift from L. Kedzierski; [23]), and arginase (generous gift from B. Ullman; [24]) were carried out as described previously [2], [4].

### Immunofluorescence assay

Immunofluorescence assay was performed with wild-type parasites expressing pXG.HV-LmDAT-ΔC_3_ as described previously [16]. The recombinant His_6_-V5 (HV) tagged HV-*Lm*DAT-ΔC_3_ was revealed with V5 monoclonal antibodies (Invitrogen) and hypoxanthine guanine phosphoribosyltransferase was visualized with specific rabbit polyclonal immunoglobulins [22]. Both antibodies were used at a 1:500 dilution. Images were taken with a Leica fluorescence microscope.

## Results and Discussion

### The N-terminal extension is important for *Lm*DAT activity

**Figure 2:**
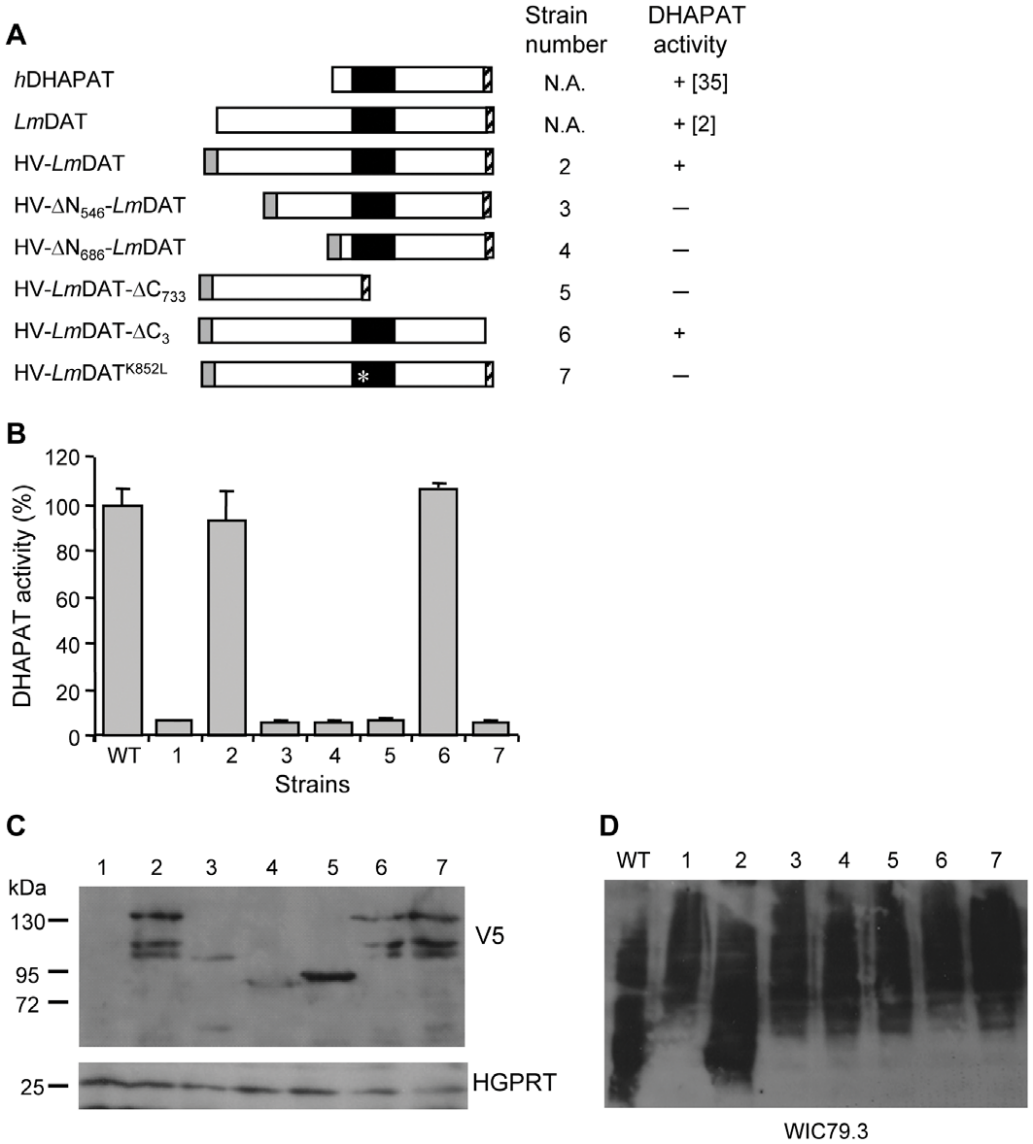
Characterization of mutant forms of *Lm*DAT. (A) Schematic representation of human DHAPAT (*h*DHAPAT) and mutant forms of *Lm*DAT. The grey rectangle, the black rectangle and the hatched area depict the HV tag, the conserved domain, and the C-terminal glycosomal targeting tripeptide, respectively, and the asterisk depicts the point mutation. B) DHAPAT activity was quantified as described in Materials and Methods. Equivalent of 0.5 mg protein extracts were applied for the assay. Null mutant alone or expressing HV-tagged wild-type and mutant forms of *Lm*DAT were used as a source of protein extracts. Activity is expressed as percentage of the positive control, the wild type (WT). The assay was performed twice in duplicate, and the graph depicts one representative experiment. Standard deviations are shown. (C) Western blot analyses in the presence of V5-specific (upper; V5) and hypoxanthine guanine phosphoribosyltransferase specific (lower; HGPRT; loading control) antibodies. Equivalent of 5×10^7^ cells were loaded in each lane. The apparent molecular weight is shown on the left. (D) Western blot analysis in the presence of WIC79.3 antibody to detect LPG. Equivalent of 10^6^ cells were loaded in each lane. (B, C, D): 1, *Δlmdat/Δlmdat*; 2, *Δlmdat/Δlmdat [HV-LmDAT NEO]*; 3, *Δlmdat/Δlmdat [HV-ΔN_546_-LmDAT NEO]*; 4, *Δlmdat/Δlmdat [HV-ΔN_686_-LmDAT NEO]*; 5, *Δlmdat/Δlmdat [HV-LmDAT-ΔC_733_ NEO]*; 6, *Δlmdat/Δlmdat [HV-LmDAT-ΔC_3_ NEO]*; 7, *Δlmdat/Δlmdat [HV-LmDAT^K852L^ NEO]*; WT, wild type.

Amino acid sequence alignment of DHAPAT enzymes from human, rat, *Caenorhabditis elegans, Trypanosoma brucei* and *Trypanosoma cruzi* shows that orthologs of parasites of the trypanosomatidae family bear a very large N-terminal extension of approximately 650 amino acids that is absent in higher eukaryotic orthologs ([16]; Fig. 2A; data not shown). Curiously, this domain fails to exhibit any similarity to known proteins and thus, is parasite specific. The function of this N-terminal extension was first investigated by creating truncated *Lm*DAT versions that were N-terminally tagged with a hexahistidine fused to a V5 epitope (HV) for visualization with the V5-specific monoclonal antibody. Plasmids coding for truncated proteins lacking the N-terminal 546 and 686 amino acids (HV-ΔN_546_-*Lm*DAT and HV-ΔN_686_-*Lm*DAT), respectively, were constructed, as well as a plasmid coding for a recombinant protein missing the 733 C-terminal amino acids (HV-*Lm*DAT-ΔC_733_). In the latter case, the *Lm*DAT C-terminal tripeptide SKM was fused to the C-terminal portion of the truncated protein to ensure glycosomal targeting [25], [26], [27]. These recombinant proteins, as well as a wild-type HV tagged version (HV-*Lm*DAT), were expressed in the null mutant *Δlmdat/Δlmdat* background [16].

**Figure 3:**
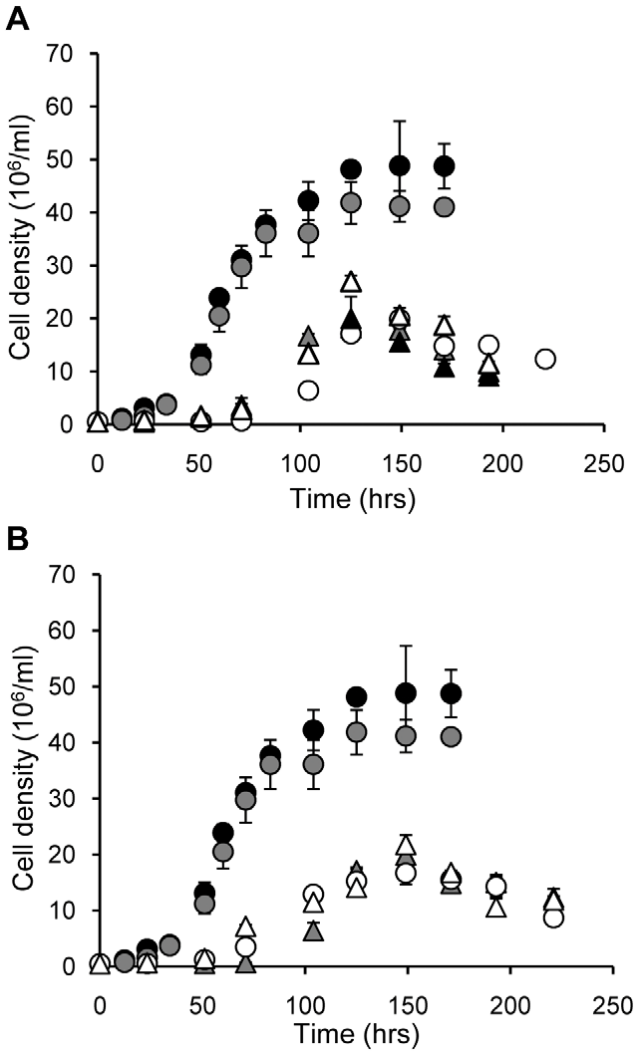
Growth curves. Cells were inoculated at a cell density of 5×10^5^/ml and were enumerated with a hemacytometer as a function of time. The assay was performed twice and the graphs represent a typical experiment. Standard deviations are shown. (A) Black circles, wild type; grey circles, complemented line *Δlmdat*/*Δlmdat [HV-LmDAT NEO]*; white circles, *Δlmdat*/*Δlmdat*; white triangles, *Δlmdat/Δlmdat [HV-ΔN_546_-LmDAT NEO]*; grey triangles, *Δlmdat/Δlmdat [HV-ΔN_686_-LmDAT NEO]*; black triangles, *Δlmdat/Δlmdat [HV-LmDAT-ΔC_733_ NEO]*. (B) Black circles, wild type; grey circles, complemented line *Δlmdat*/*Δlmdat [HV-LmDAT NEO]*; white circles, *Δlmdat*/*Δlmdat*; white triangles, *Δlmdat*/*Δlmdat [HV-LmDAT-ΔC_3_ NEO]*; grey triangles, *Δlmdat*/*Δlmdat [HV-LmDAT^K852L^ NEO]*.

**Figure 4:**
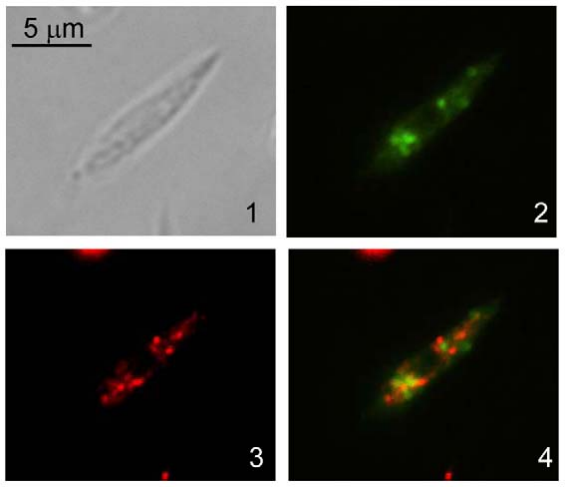
HV-*Lm*DAT-ΔC_3_ does not localize in the glycosomes. Wild type expressing recombinant HV-*Lm*DAT-ΔC_3_ was analyzed by phase contrast (panel 1) or immunofluorescence microscopy using anti-V5 antibody (panel 2) or polyclonal antiserum specific to hypoxanthine guanine phosphoribosyltransferase (panel 3). Panel 4 shows the merge of panels 2 and 3.

We verified first that the HV tag did not affect the function of *Lm*DAT; the recombinant protein HV-*Lm*DAT was expressed as an approximately 150 kDa band as shown by Western blot analysis using the monoclonal antibody specific to the V5 epitope (Fig. 2C). Bands present at lower molecular weights very likely represent degradation products of the full length tagged protein. In addition, HV-*Lm*DAT was enzymatically active, and supported normal growth and survival during the stationary phase (Fig. 2B, 3A; [16]). The presence of ether lipids was assessed by investigating the migration behavior of the ether lipid anchored virulence factor LPG; it has been established that *Leishmania* mutants lacking ether lipids synthesize slow migrating forms of LPG as a result of hyperglycosylation of its disaccharide domain [2], [4]. Western blot analysis showed that HV-*Lm*DAT restored expression of normal migrating forms of LPG while the null mutant expressed slower migrating LPG species (Fig. 2D; [4]).

Deletions of the N-terminal (first 546 or 686 amino acids) but not of the C-terminal 733 amino acids led to low intensity signals as shown by Western blot analysis using the monoclonal antibody specific to the V5 epitope, as the corresponding bands at apparent weights of approximately 100 and 83 kDa respectively, were hardly detectable (Fig. 2C). Accordingly, none of the N-terminal (HV-ΔN_546_-*Lm*DAT and HV-ΔN_686_-*Lm*DAT) and C-terminal (HV-*Lm*DAT-ΔC_733_) truncated proteins rescued the slow growth or the rapid death during the stationary phase (Fig. 3A). Consistent with these results, no DHAPAT activity could be measured with cells expressing any of these truncated versions of *Lm*DAT (Fig. 2B). Accordingly, Western blot analyses in the presence of WIC79.3 for detection of LPG revealed that null mutant strains expressing any of the three truncated forms of *Lm*DAT made glycolipids migrating slower than that of the wild type but similar to that of the null mutant (Fig. 2D; [4]).

Our results show that N-terminal truncated versions of *Lm*DAT gave lower intensity signals in Western blot analysis in the presence of anti-V5 antibody. A possible explanation is that the V5 antibody has lower affinity for these recombinant proteins or less access to the epitope due to conformation issues. In this case the N-terminal domain may indirectly be important for catalysis; because this domain is absent in higher eukaryotic counterparts (Fig. 2A; data not shown; [16]), it is doubtful that it is directly involved in substrate recognition or catalysis. These events are expected to occur in the C-terminal domain of *Lm*DAT that is shared by all DHAPAT orthologs (Fig. 2A; [16]) and this assumption is corroborated by the fact that HV-*Lm*DAT-ΔC_733_ is inactive. Another possible interpretation is that the N-terminal domain somehow helps this very large protein to fold properly in order to yield a stable enzyme, and thus, affects *Lm*DAT activity indirectly. All together these data suggest that designing a compound specific to the N-terminal extension may represent a reasonable strategy to inactivate *Lm*DAT.

### A catalytically active *Lm*DAT enzyme is necessary for growth and survival during the stationary phase

In contrast to *Δlmdat/Δlmdat*, the *Δads1/Δads1* mutant, carrying a genetic deletion of the alkyl DHAP synthase gene and lacking ether lipids, does not exhibit any growth phenotype, suggesting that ether lipids are dispensable for growth and survival during the stationary phase [2]. Thus, *Lm*DAT may be a bipartite protein with an N-terminal domain involved in growth and survival during the stationary phase, and a C-terminal domain functioning in catalysis. To assess whether a catalytically active *Lm*DAT DHAPAT is important for normal growth and survival during the stationary phase, a mutant enzyme was created that bears a leucine instead of a lysine at position 852 (K852L), based on the information that replacement of the corresponding conserved amino acid by a histidine in the human DHAPAT abrogated its enzymatic activity [28]. As described above, a HV-tagged version (HV-*Lm*DAT^K852L^) was expressed in the null mutant background. Western blot analysis was performed to verify its expression level that was similar to HV-tagged wild-type *Lm*DAT (Fig. 2C). HV-*Lm*DAT^K852L^ enzyme displayed no DHAPAT activity and led to the production of slow migrating LPG glycolipids similar to that of the null mutant (Fig. 2B, 2D). In addition, HV-*Lm*DAT^K852L^ failed to support normal growth and survival during the stationary phase (Fig. 3B).

Altogether, these results demonstrate that the conserved lysine K852 is important for substrate recognition or catalysis of *Lm*DAT similar to the human ortholog. Together with the fact that the N-terminal domain alone is unable to support normal growth and survival during the stationary phase, our results suggest that the acyltransferase activity of *Lm*DAT itself is critical for normal growth and survival during the stationary phase. Our data also exclude the idea that *Lm*DAT is a bipartite enzyme with an N-terminal domain responsible for growth and/or survival during the stationary phase, and a C-terminal part implicated in acyltransferase activity.

### Glycosomal expression of *Lm*DAT is critical for LPG synthesis

*Lm*DAT resides in glycosomes consistent with the presence of a typical type 1 C-terminal glycosomal targeting signal sequence (SKM), that is sufficient for targeting proteins to this organelle [16], [19]. Hence, the importance of the glycosomal subcellular localization of *Lm*DAT for ether lipid biosynthesis was assessed by expressing a HV-tagged *Lm*DAT recombinant enzyme lacking the C-terminal glycosomal targeting tripeptide SKM (HV-*Lm*DAT-ΔC_3_) in the null mutant background [25], [27], [29]. Western blot analysis was performed in the presence of anti-V5 monoclonal antibodies demonstrated that the levels of HV-*Lm*DAT-ΔC_3_ protein were similar to that of wild-type tagged HV-*Lm*DAT expressed in the null mutant background (Fig. 2C).

**Figure 5:**
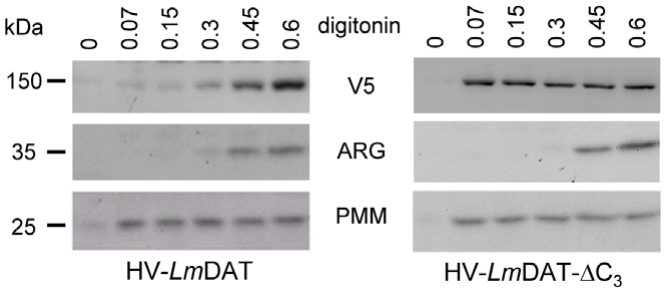
Digitonin fractionation followed by Western blot analysis. FV1 *[HV-LmDAT NEO]* and FV1 *[HV-LmDAT-ΔC_3_ NEO]* were fractionated in the presence of digitonin as described in Materials and Methods. Cell supernatants were then subjected to Western blot analysis in the presence of monoclonal anti-V5 antibodies (V5), and of polyclonal immunoglobulins specific to arginase (ARG) and phosphomannomutase (PMM). Equivalent of 10^7^ cell supernants were loaded in each lane. The apparent molecular weight markers are shown.

Subcellular localization of HV-*Lm*DAT-ΔC_3_ was assessed by immunofluorescence assay. HV-*Lm*DAT-ΔC_3_ was revealed in the presence of V5 monoclonal antibodies and gave a signal that was partially overlapping with that obtained with antibodies specific to the glycosomal resident hypoxanthine guanine phosphoribosyltransferase suggesting a glycosomal association ([22]; Fig. 4). However, digitonin fractionation provided evidence for HV-*Lm*DAT-ΔC_3_ localizing outside the glycosomes. Digitonin specifically permeabilizes the cytoplasmic membrane at low concentrations while high doses of this detergent are needed to solubilize the glycosomal membrane [30]. HV-*Lm*DAT required a minimal concentration of digitonin of 0.45 mg/ml in order to be properly released in the cell supernatant and behaved similarly as the glycosomal resident arginase ([31]; Fig. 5), In contrast, HV-*Lm*DAT-ΔC_3_ readily fractionated in the cell supernant at a low digitonin concentration of 0.075 mg/ml, as the cytosolic enzyme phosphomannomutase [23]. Our data suggest that HV-*Lm*DAT-ΔC_3_ is partially associated with glycosomes and possibly other organelles but resides on the cytosolic side of the organellar membrane. This is consistent with previous studies that demonstrated that abrogation of the type 1 C-terminal glycosomal targeting signal sequence resulted in a cytoplasmic localization [25], [27], [29].

*In vitro* DHAPAT assays showed that HV-*Lm*DAT-ΔC_3_ was enzymatically active as the wild-type HV-tagged version of *Lm*DAT (Fig. 2B), demonstrating that the C-terminal glycosomal targeting sequence is dispensable for enzymatic activity. Surprisingly, Western blot analysis performed in the presence of WIC79.3 demonstrated that, in contrast to HV-*Lm*DAT, HV-*Lm*DAT-ΔC_3_ expression failed to restore the synthesis of normal migrating LPG (Fig. 2D). Consistent with this result, expression of HV-*Lm*DAT-ΔC_3_ did not ameliorate the slow growth and survival during the stationary phase of the null mutant (Fig. 3B). These data suggest that the proper acyl donor for *Lm*DAT, palmitoyl-CoA, may not be available in the cytosol [16]. This is unlikely because fatty acyl-CoAs are made either in the endoplasmic reticulum by elongases or in the mitochondria by type II fatty acyl-CoA synthases, and have to be transported *via* the cytosol to the endoplasmic reticulum and glycosomes where lipid biosynthesis occurs [16], [32], [33], [34]. Alternatively, the role of the glycosomal compartmentalization of *Lm*DAT is to sequester its product, 1-acyl-DHAP, in this organelle for conversion into 1-alkyl-DHAP by the glycosomal alkyl DHAP synthase ADS1 rather than being metabolized to 1-acyl-G3P by the cytosolic alkyl/acyl-DHAP reductase for the synthesis of ester glycerolipids [2], [17]. Last, DHAP produced in the glycosome may not be available in the cytosol where HV-*Lm*DAT-ΔC_3_ accumulates [19]. These results are also in accordance with the idea that the glycosomal membrane does not allow transport of 1-acyl-DHAP from the cytosol into the glycosome for conversion to 1-alkyl-DHAP by the alkyl-DHAP synthase ADS1.

## References

[1] Desjeux P (2004). Leishmaniasis: current situation and new perspectives.. Comp Immunol Microbiol Infect Dis.

[2] Zufferey R, Allen S, Barron T, Sullivan DR, Denny PW (2003). Ether phospholipids and glycosylinositolphospholipids are not required for amastigote virulence or for inhibition of macrophage activation by *Leishmania major*.. J Biol Chem.

[3] Beach DH, Holz GG, Anekwe GE (1979). Lipids of *Leishmania* promastigotes.. J Parasitol.

[4] Zufferey R, Al-Ani GK, Dunlap K (2009). *Leishmania* dihydroxyacetonephosphate acyltransferase *Lm*DAT is important for ether lipid biosynthesis but not for the integrity of detergent resistant membranes.. Mol Biochem Parasitol.

[5] Wassef MK, Fioretti TB, Dwyer DM (1985). Lipid analyses of isolated surface membranes of *Leishmania donovani* promastigotes.. Lipids.

[6] Descoteaux A, Turco SJ (2002). Functional aspects of the *Leishmania donovani* lipophosphoglycan during macrophage infection.. Microbes Infect.

[7] Ilg T (2001). Lipophosphoglycan of the protozoan parasite *Leishmania*: stage- and species-specific importance for colonization of the sandfly vector, transmission and virulence to mammals.. Med Microbiol Immunol (Berl).

[8] Matlashewski G (2001). *Leishmania* infection and virulence.. Med Microbiol Immunol (Berl).

[9] Naderer T, Vince JE, McConville MJ (2004). Surface determinants of *Leishmania* parasites and their role in infectivity in the mammalian host.. Curr Mol Med.

[10] Sacks DL (2001). *Leishmania*-sand fly interactions controlling species-specific vector competence.. Cell Microbiol.

[11] Schneider P, Ferguson MA, McConville MJ, Mehlert A, Homans SW (1990). Structure of the glycosyl-phosphatidylinositol membrane anchor of the *Leishmania major* promastigote surface protease.. J Biol Chem.

[12] Ilgoutz SC, McConville MJ (2001). Function and assembly of the *Leishmania* surface coat.. Int J Parasitol.

[13] Ferguson MA (1997). The surface glycoconjugates of trypanosomatid parasites.. Philos Trans R Soc Lond B Biol Sci.

[14] McConville MJ, Turco SJ, Ferguson MA, Sacks DL (1992). Developmental modification of lipophosphoglycan during the differentiation of *Leishmania major* promastigotes to an infectious stage.. Embo J.

[15] Zhang K, Beverley SM Phospholipid and sphingolipid metabolism in *Leishmania*.. Mol Biochem Parasitol.

[16] Zufferey R, Ben Mamoun C (2006). *Leishmania major* expresses a single dihydroxyacetone phosphate acyltransferase localized in the glycosome, important for rapid growth and survival at high cell density and essential for virulence.. J Biol Chem.

[17] Heise N, Opperdoes FR (1997). The dihydroxyacetonephosphate pathway for biosynthesis of ether lipids in *Leishmania mexicana* promastigotes.. Mol Biochem Parasitol.

[18] Zufferey R, Mamoun CB (2005). The initial step of glycerolipid metabolism in *Leishmania major* promastigotes involves a single glycerol-3-phosphate acyltransferase enzyme important for the synthesis of triacylglycerol but not essential for virulence.. Mol Microbiol.

[19] Opperdoes FR, Borst P (1977). Localization of nine glycolytic enzymes in a microbody-like organelle in *Trypanosoma brucei*: the glycosome.. FEBS Lett.

[20] Ngo H, Tschudi C, Gull K, Ullu E (1998). Double-stranded RNA induces mRNA degradation in *Trypanosoma brucei*.. Proc Natl Acad Sci U S A.

[21] Kelleher M, Bacic A, Handman E (1992). Identification of a macrophage-binding determinant on lipophosphoglycan from Leishmania major promastigotes.. Proc Natl Acad Sci U S A.

[22] Shih S, Hwang HY, Carter D, Stenberg P, Ullman B (1998). Localization and targeting of the *Leishmania donovani* hypoxanthine-guanine phosphoribosyltransferase to the glycosome.. J Biol Chem.

[23] Garami A, Mehlert A, Ilg T (2001). Glycosylation defects and virulence phenotypes of *Leishmania mexicana* phosphomannomutase and dolicholphosphate-mannose synthase gene deletion mutants.. Mol Cell Biol.

[24] Carter NS, Yates PA, Gessford SK, Galagan SR, Landfear SM (2010). Adaptive responses to purine starvation in *Leishmania donovani*.. Mol Microbiol.

[25] Sommer JM, Peterson G, Keller GA, Parsons M, Wang CC (1993). The C-terminal tripeptide of glycosomal phosphoglycerate kinase is both necessary and sufficient for import into the glycosomes of *Trypanosoma brucei*.. FEBS Lett.

[26] Sommer JM, Cheng QL, Keller GA, Wang CC (1992). *In vivo* import of firefly luciferase into the glycosomes of *Trypanosoma brucei* and mutational analysis of the C-terminal targeting signal.. Mol Biol Cell.

[27] Sommer JM, Wang CC (1994). Targeting proteins to the glycosomes of African trypanosomes.. Annu Rev Microbiol.

[28] Ofman R, Hettema EH, Hogenhout EM, Caruso U, Muijsers AO (1998). Acyl-CoA:dihydroxyacetonephosphate acyltransferase: cloning of the human cDNA and resolution of the molecular basis in rhizomelic chondrodysplasia punctata type 2.. Hum Mol Genet.

[29] Blattner J, Swinkels B, Dorsam H, Prospero T, Subramani S (1992). Glycosome assembly in trypanosomes: variations in the acceptable degeneracy of a COOH-terminal microbody targeting signal.. J Cell Biol.

[30] Jardim A, Rager N, Liu W, Ullman B (2002). Peroxisomal targeting protein 14 (PEX14) from *Leishmania donovani*. Molecular, biochemical, and immunocytochemical characterization.. Mol Biochem Parasitol.

[31] Roberts SC, Tancer MJ, Polinsky MR, Gibson KM, Heby O (2004). Arginase plays a pivotal role in polyamine precursor metabolism in *Leishmania*. Characterization of gene deletion mutants.. J Biol Chem.

[32] Lee SH, Stephens JL, Englund PT (2007). A fatty-acid synthesis mechanism specialized for parasitism.. Nat Rev Microbiol.

[33] Stephens JL, Lee SH, Paul KS, Englund PT (2007). Mitochondrial fatty acid synthesis in *Trypanosoma brucei*.. J Biol Chem.

[34] Lee SH, Stephens JL, Paul KS, Englund PT (2006). Fatty acid synthesis by elongases in trypanosomes.. Cell.

[35] Ofman R, Hettema EH, Hogenhout EM, Caruso U, Muijsers AO (1998). Acyl-CoA:dihydroxyacetonephosphate acyltransferase: cloning of the human cDNA and resolution of the molecular basis in rhizomelic chondrodysplasia punctata type 2.. Hum Mol Genet.

